# Vascular Endothelial Growth Factor Levels in Dobrava/Belgrade Virus Infections

**DOI:** 10.3390/v5123109

**Published:** 2013-12-10

**Authors:** Katerina Tsergouli, Anna Papa

**Affiliations:** Department of Microbiology, Medical School, Aristotle University of Thessaloniki, Thessaloniki 54124, Greece; E-Mail: ktsergouli@gmail.com

**Keywords:** vascular endothelial growth factor, Dobrava/Belgrade virus, hemorrhagic fever with renal syndrome, hantavirus, Greece

## Abstract

The levels of vascular endothelial growth factor-A (VEGF) were estimated in 102 serum samples from 63 hospitalized Greek patients with hemorrhagic fever with renal syndrome (HFRS) caused by Dobrava/Belgrade virus. Significantly higher VEGF levels were seen in the severe when compared with non-severe cases (mean values 851.96 pg/mL and 326.75 pg/mL, respectively; *p* = 0.003), while a significant difference was observed among groups based on the day after the onset of illness. In both severe and non-severe cases, VEGF peaked in the second week of illness; however, elevation of VEGF in the severe cases started later and remained high until convalescence, suggesting that the role of VEGF was associated with repair of vascular damage rather than with increased permeability.

## 1. Introduction

Hantaviruses (genus *Hantavirus*, Family *Bunyaviridae*) are transmitted to humans mainly by the inhalation of aerosolized excreta of infected rodents and cause in humans hemorrhagic fever with renal syndrome (HFRS) in Asia and Europe, and hantavirus pulmonary syndrome (HPS) in the Americas [1]. Dobrava/Belgrade virus (DOBV), especially the one associated with the rodent *Apodemus flavicollis*, causes a severe form of HFRS with fatality rate up to 10% [2,3,4]. A retrospective genetic study in Greece showed that the responsible virus for all the PCR-positive HFRS cases observed during a 17-year period was DOBV, and the Greek DOBV sequences cluster together with respective sequences obtained from *A. flavicollis* [5].

HFRS is characterized by acute renal failure with often massive proteinuria caused by tubular and glomerular involvement [6]. After an incubation period of approximately 2–4 weeks, HFRS patients present a febrile, flu-like syndrome lasting 3–7 days, which is followed by hypotensive (a few hours to two days), oliguric (3–7 days) and diuretic (1–2 weeks) phases, leading to convalescence [7]. Hemorrhagic manifestations may appear towards the end of the febrile phase, while renal failure occurs during the hypotensive phase; pulmonary involvement is present in several cases, and sometimes acute respiratory distress syndrome (ARDS) develops [7,8].

Hantaviruses infect endothelial cells and induce capillary permeability. The integrity and function of vascular endothelial and glomerular barriers are maintained by both tight and adherens junctions [9,10]. Because hantaviruses are not cytopathic for endothelial cells, illness appears to result from immunopathological mechanisms involving innate and adaptive immune responses [11]. Vascular endothelial growth factors (VEGFs) are key regulators of permeability [12]. Among the 5 VEGFs, VEGF-A is one of the most potent vascular permeability agents known (originally described as vascular permeability factor), produced by various cell types (including endothelial, glomerular epithelial, and tubular cells), and stimulates vasculogenesis and angiogenesis following its binding with tyrosine kinase receptors (VEGFRs) [12]. It is part of the system that restores the oxygen supply to tissues when blood circulation is inadequate. Pathogenic hantaviruses bind to αvβ3 integrins and markedly increase the permeability of endothelial cells in response to VEGF, whereas non-pathogenic hantaviruses have not such effect [13,14,15,16]. Specifically, pathogenic hantaviruses induce increased phosphorylation of the VEGFR2 in infected endothelial cells, which leads to phosphorylation, internalization and degradation of vascular endothelial cadherin (VE-cadherin), predominant structural component of the adherens junctions, resulting in paracellular permeability and microvascular leak [17].

Aim of the present study was to investigate the role of VEGF in DOBV infections, by estimating its serum levels in laboratory confirmed HFRS cases in various stages of the disease.

## 2. Results and Discussion

### 2.1. Grouping of HFRS Cases

The total of 102 serum samples collected during 1994–2012 from 63 hospitalized HFRS Greek patients (56 males/7 females, aged 11–73 years, median age 35 years) were divided into five groups according to the day after onset of the symptoms: group A included 24 samples from 24 HFRS patients during the first week of illness; group B, 43 samples from 37 patients during the second week; group C, 23 samples from 22 patients during the third week; group D, 6 samples from 6 patients taken during the fourth week; and group E included 6 samples from 5 patients taken after the 28th day of illness (28–70 days). Serum samples from 21 apparently healthy blood donors (13 males/8 females), aged 21–45 years (median 34 years) were included in the study as control group. Among the 63 cases, 32 were considered as severe since 22 presented with hemorrhagic manifestations, 9 with symptoms from the respiratory system (two of them ARDS), 2 with sepsis, 5 were admitted to ICU, and 14 underwent hemodialysis. Two cases had a fatal outcome.

### 2.2. Estimation of VEGF Levels

**Figure 1:**
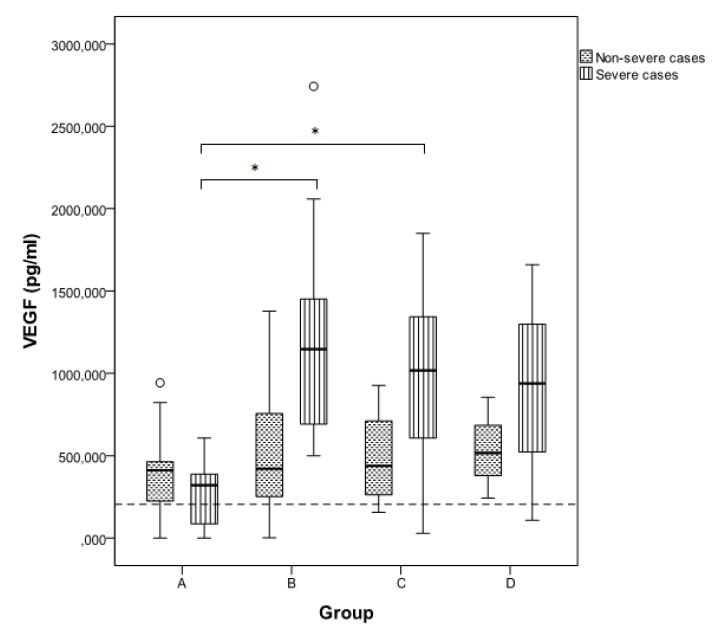
Comparison of VEGF levels between severe and non-severe cases in groups A–D. Data are given as median (thick line), 25th–75th percentile (box) and range (whiskers). Significant differences between groups (*p* < 0.05) are shown with an asterisk. Group E is not shown, as only one sample was available from non-severe cases. The dotted line is set to 204.03 pg/mL and represents the mean VEGF value of the control group.

Serum VEGF levels in the 102 samples of the 63 HFRS patients ranged from 0.00 to 2742.00 pg/mL (mean 666.22, S.D. 532.64), and were significantly increased compared to those of the control group (mean 204.03, S.D. 120.22) (*p* < 0.05). Comparing with the control group, VEGF level was significant higher in the severe and non-severe cases in all five groups (*p* < 0.05), except the severe cases in group A (first week), in which VEGF level did not differ significantly (*p* = 0.140). In total, VEGF levels were significantly higher in the severe than in non-severe cases (mean values 851.96 pg/mL and 326.75 pg/mL, respectively; *p* = 0.003) (Table 1). Specifically, among the five groups, a significant difference between severe and non-severe cases was seen in group B (*p* < 0.001) and group C (*p* = 0.026) (Table 1). Significant increase of VEGF levels among severe cases was observed between groups A and B, and A and C (*p* < 0.001 and 0.001, respectively) (Figure 1), while VEGF decreased significantly in the fifth week of illness (2nd *vs*. 5th week *p* = 0.025); however, the mean level in the 5th week (504.07 pg/mL) was still more than two fold the mean value of the control group. In non-severe cases, VEGF started to increase earlier, peaked in the second week of illness, remained elevated in the third week and decreased afterwards, with no significant differences among groups (*p* > 0.05). Especially in group A (first week of illness, febrile phase), VEGF levels were higher in the non-severe than in severe cases, while in all other groups VEGF was higher in the severe cases. In both severe and non-severe cases, higher levels were observed during and after the second week of illness, suggesting that the increased VEGF might be associated with repair of the vascular damage rather than with increased permeability. The higher VEGF levels in the severe cases can be explained by the fact that the damage in these cases was greater and prompted for increased VEGF release in order to act on the endothelium and stimulate the vascular remodeling and growth.

**Table 1:** Range and mean vascular endothelial growth factor-A (VEGF) levels (in pg/mL) in serum samples taken from severe and non severe hemorrhagic fever with renal syndrome (HFRS) cases grouped according to the day after the onset of illness: Group A: 1st week, B: 2nd week, C: 3rd week, D: 4th week, E: >4 weeks.

Group (n)	Severe			Non severe			*p*-value
	N	Range	Mean (SD)	N	Range	Mean (SD)	
A (24)	11	0.00–607.23	267.16 (201.11)	13	0.00–942.44	391.06 (303.67)	0.338
B (43)	22	499.53–2742.00	1159.64 (582.47)	21	1.07–1378.00	514.89 (383.59)	<0.001
C (23)	10	28.76–1850.30	977.37 (528.65)	13	156.46–927.23	508.74 (280.28)	0.026
D (6)	3	107.20–1660.00	901.73 (777.03)	3	242.44–854.00	537.48 (306.34)	0.513
E (6)	5	90.30–1742.00	504.07 (694.68)	1	382.00	382.00	0.380
Total (102)	51	0.00–2742.00	851.96 (629.23)	51	0.00–1378.00	480.48 (326.75)	0.003

A strong correlation was observed between VEGF levels and severity of the disease (r_s_ = 0.350, *p* < 0.001), presence of hemorrhagic manifestations (r_s_ = 0.321, *p* = 0.001), pulmonary involvement (r_s_ = 0.220, *p* = 0.026), and need for renal dialysis (r_s_ = 0.251, *p* = 0.011). This finding, together with the time when VEGF was elevated, suggests that the increased VEGF release was in response to the increased demand for tissue repair. Similarly, when plasma VEGF levels were evaluated during dengue infection, it was found that they were significantly higher in patients with dengue hemorrhagic fever than in dengue fever patients [18].

Increased VEGF levels were seen in the two fatal cases. For one of the fatal cases (100/97), 3 serial samples were available, and VEGF was steadily high (1291.33 pg/mL, 1020.22 pg/mL and 938 pg/mL in samples taken on 13th, 16th and 22nd day of illness, respectively). One sample taken on the 10th day of illness was available for the second fatal case (VEGF 691.33 pg/mL). However, since the number of fatal cases was low, it is not possible to reach to a conclusion on the correlation between VEGF and fatal outcome.

The VEGF dynamic patterns were more obvious when serial samples were tested (Table 2). Especially in one of the patients with ARDS and sepsis (38/02) for whom two serial samples were available, VEGF levels were elevated at the same scale on the 22nd and 33rd day of illness (1660 pg/mL and 1742 pg/mL, respectively), probably consistent with vascular repair. The relatively low VEGF levels during the first week of illness in the severe cases suggests that VEGF is not the main factor related to permeability. This fact was shown recently in a capillary blood vessel-like *in vitro* system, in which the infected vessels neither lost integrity nor displayed evidence of VE-cadherin degradation, despite the presence of VEGF; in contrast, there was evidence of a novel mechanism of hantavirus-induced vascular leakage involving factor XII-dependent activation of the plasma kallikrein-kinin system and liberation of bradykinin [19].

Earlier VEGF increase in mild than in severe cases was also seen in HFRS cases caused by Hantaan virus (HTNV), the prototype hantavirus; the elevation of VEGF began from the onset of fever, increased gradually, with the peak level in mild cases being observed at the oliguric stage, whereas in the moderate or severe groups the peak level was observed later, at the diuretic stage, with VEGF levels at convalescent stage being positively correlated with the degree of the disease severity [20]. The authors reported that the high level of serum VEGF at the oliguric stage may be involved in the pathogenesis of renal injury, but the sustained high level of serum VEGF at diuretic or convalescence stage would probably contribute to the renal recovery after the clearance of the virus [20], thus, suggesting a dual role for VEGF, depending on the stage of the disease. In contrast, in another study on HFRS cases caused by HTNV, the differences between control subjects and patients in the diuretic phase or in convalescence were not significant [21]. This controversy could be explained if the tested cases of the second study were not severe.

Similar to the results of the present study in HFRS cases, were those in HPS cases: during the first three days of hospitalization, the circulating serum and plasma VEGF levels in severe cases were low, and an increase was seen during recovery (11–20 days after-admission), while VEGF levels in mild and severe HPS patients were 2 to >3 fold higher, respectively, than in controls [22]. However, localized VEGF responses are directly involved in acute HPS pathogenesis, since high VEGF levels had been detected in pulmonary edema fluid and peripheral blood mononuclear cells during acute HPS stages [22]. This data is supported by *in vitro* studies demonstrating that VEGF is involved in the loss of endothelial barrier function, since it was shown that pathogenic hantaviruses disrupt fluid barrier properties of endothelial cell adherens junctions by enhancing VEGFR2-VE-cadherin pathway responses which increase paracellular permeability [11,16,23]. In a previous study it was shown that an antibody that blocks VEGFR2 activation is able to block internalization of VE-cadherin in cells infected by Andes hantavirus (ANDV), suggesting that compounds that target the interactions of VEGF, VE-cadherin and αvβ3 integrins could be a potential approach for therapeutic interventions [24]. In the same study, higher VEGF levels were detected in 9 HPS patients with ARDS than 6 patients with other respiratory infection [24]. A recent study in the lethal hamster model of HPS showed that VEGF upregulation was not observed in plasma of ANDV-infected hamsters [25], while experimental ANDV infection in deer mice (heterologous rodent host) showed that although they mounted a humoral immune response, they didn’t show any clinical signs or histopathological changes [26]. Results of all these studies show that there are differences in local *versus* systemic VEGF responses, and in acute *versus* convalescent stage of the disease, while important role plays also the associated hantavirus strain. The age and gender parameters were not analyzed in the present study, since a male-biased incidence of HFRS is observed in Greece, and most of the patients were <40 years old, reflecting that this specific group of the population is involved in agricultural and husbandry activities, thus being at increased risk for acquiring hantavirus infection.

**Table 2:** Serum VEGF levels in 32 patients with DOBV infection for whom serial samples were available. A. severe cases, B. non-severe cases. The day of illness, and data about hospitalization in intensive care unit (ICU), need of dialysis, presence of hemorrhagic manifestations, and pulmonary involvement are shown. ARDS: acute respiratory distress syndrome. DIC: Disseminated intravascular coagulation.

Patient ID	Age	Sex	ICU/hemorrhagic manifestations/pulmonary involvement/dialysis	Group A (1st week)		Group B (2nd week)		Group C (3rd week)		Group D (4th week)		Group E (>4th week)	
				Day	VEGF (pg/mL)	Day	VEGF (pg/mL)	Day	VEGF (pg/mL)	Day	VEGF (pg/mL)	Day	VEGF (pg/mL)
			**A. Severe cases**										
167/05	29	M	ICU, dialysis	5	0.00			17	1545.69				
108/07	23	F	ICU, hemorrhages, pulmonary edema	7	359.53	12	1678.00						
53/10	51	F	hemodialysis			9	771.43	16	1100.00			35 70	234.29 90.30
102/04	40	M	pulmonary infiltrations, hematuria, dialysis			10	508.14					27	107.20
184/05	44	M	dialysis, hematuria			8 11 13	2742.00 2058.57 1910.00						
188/06	38	M	dialysis, hematuria			12	1050.30	17	607.23				
79/02	28	M	petechiae, hemoptysis	6	342.00	8	634.00						
43/02	27	M	dialysis			9	534.00	15	1014.00				
38/02	35	M	ARDS, sepsis, dialysis, hemorrhages							22	1660.00	33	1742.00
385/01	20	M	hematuria			10	708	21	262				
162/98	59	M	hematuria	7	0	13	1242.44						
85/97	70	M	sepsis	7	415.77	10	1029.11						
100/97	38	M	ICU, fatal			13	1291.33	16	1020.22	22	938.00		
62/02	36	M	Pulmonary involvement, dialysis, DIC			8 9 10	1318,00 1289.11 1344.66						
177/06	31	M	ARDS			11	1480.22	17	1342.44				
			**B. Non-severe cases**										
58/10	40	M		6	743.84			15	491.84				
116/07	33	F		7	27.23			15	278.00				
159/07	21	M		7	0.00	15	824.66						
196/07	35	M		7	262.86	12	490.00						
236/05	22	M				8 11	1236.46 376.46						
62/10	29	M		7	413.38	11	242.44			26	1.07		
240/04	32	M				8 11	962.61 1062.61						
66/03	49	M				10	48.00	16	156.46				
252/04	38	F						16 18	927.23 913.55				
18/02	19	M						17	626.00	25	516.00		
34/09	38	M				14	755.77	19	211.33				
243/95	35	F		5	822.44			16	664.66				
218/95	42	M		6	411.33	13	182,44						
258/95	40	M		6	426.88	10	251.33						
254/96	24	M		5	344.66	13	355,77						
28/96		M				10	835.77	17	711.33				
278/00	21	M						16	438.00	22	854.00	30	382.00

## 3. Experimental Section

### 3.1. ELISA

Serum VEGF levels were measured using sandwich enzyme-linked immunosorbent assay (ELISA) (Human VEGF-A Platinum ELISA, Bender MedSystems GmbH, Vienna, Austria) according to the instructions of the manufacturers. The reported sensitivity of the VEGF detection is >7.9 pg/mL. All samples had been stored in −70 °C; they were tested altogether, using kits with the same lot number.

### 3.2. Statistical Analysis

Statistical analysis was performed using the software package IBM SPSS Statistics version 19. For continuous variables, the Mann-Whitney *U*-test or Kruskal-Wallis test were used to evaluate the differences between groups. Spearman’s rank order correlation coefficients (r_s_) were used to calculate the strength of the relationship between two variables. Significance level was set at *p* < 0.05.

## 4. Conclusions

The present study gives a first insight of the dynamic patterns of VEGF in HFRS patients with DOBV infection. Significantly higher VEGF levels were seen in the severe rather than in non-severe cases, with the highest values being observed during and after the second week of illness, suggesting that increased VEGF might be associated with repair of the vascular damage rather than with increased permeability. However, further *in vitro* and case-control studies, especially on serial samples, from patients infected with various hantavirus strains are needed to test for signs of vascular repair in addition to VEGF levels. Since the immune response and the outcome of the disease is multifactorial, understanding the interactions of VEGF with cytokines and growth factors will elucidate the pathogenesis and pathophysiology of hantaviral infections and set the basis for therapeutic design.
